# ‘I'll be in a safe place’: a qualitative study of the decisions taken by people with advanced cancer to seek emergency department care

**DOI:** 10.1136/bmjopen-2016-012134

**Published:** 2016-11-02

**Authors:** Lesley A Henson, Irene J Higginson, Barbara A Daveson, Clare Ellis-Smith, Jonathan Koffman, Myfanwy Morgan, Wei Gao

**Affiliations:** 1 Faculty of Life Sciences & Medicine, Division of Palliative Care, Policy & Rehabilitation, King's College London, London, UK; 2 Faculty of Life Sciences & Medicine, Division of Health & Social Care Research, King's College London, London, UK

**Keywords:** End-of-life care, Cancer, Health seeking behaviour, Emergency department

## Abstract

**Objective:**

To explore the decisions of people with advanced cancer and their caregivers to seek emergency department (ED) care, and understand the issues that influence the decision-making process.

**Design:**

Cross-sectional qualitative study incorporating semistructured patient and caregiver interviews.

**Methods:**

Between December 2014 and July 2015, semistructured interviews were conducted with 18 people with advanced cancer, all of whom had recently attended the ED of a large university teaching hospital located in south-east London; and six of their caregivers. Interviews were audio recorded, transcribed verbatim and analysed using a constant comparative approach. Padgett and Brodsky's modified version of the ‘Behavioral Model of Health Services Use’ was used as a framework to guide the study.

**Results:**

Issues influencing the decision-making process included: (1) disease-related anxiety—those with greater anxiety related to their cancer diagnosis interpreted their symptoms as more severe and/or requiring immediate attention; (2) prior patterns of health-seeking behaviour—at times of crisis participants defaulted to previously used services; (3) feelings of safety and familiarity with the hospital setting—many felt reassured by the presence of healthcare professionals and monitoring of their condition; and, (4) difficulties accessing community healthcare services—especially urgently and/or out-of-hours.

**Conclusions:**

These data provide healthcare professionals and policymakers with a greater understanding of how systems of care may be developed to help reduce ED visits by people with advanced cancer. In particular, our findings suggest that the number of ED visits could be reduced with greater end-of-life symptom support and education, earlier collaboration between oncology and palliative care, and with increased access to community healthcare services.

**Strengths and limitations of this study**

- Understanding what influences people with advanced cancer to seek emergency department (ED) care is key to developing initiatives aimed at reducing high attendance; to date, however, such evidence is limited. To address this issue we conducted a qualitative interview study exploring the decision-making process of people with advanced cancer and their caregivers to seek ED care.
- Semistructured in-depth interviews were conducted with 18 people with advanced cancer, all of whom had recently attended the ED of a large university teaching hospital located in south-east London; and six of their caregivers.
- We adopted a maximum variation (heterogeneity) sampling strategy to identify people with a range of characteristics and capture potentially richer and more diverse data relevant to the research question.
- Our study interviewed people who decided to seek ED care. The decision-making process of those who used alternative services was not explored and is a limitation of this research.

## Background

A large proportion of all healthcare expenditure in developed countries is consumed by care for those in the last year of life; in the UK this is estimated at 10–20% of the National Health Service (NHS) budget, while in the USA it accounts for as much as 30% of the Medicare budget.^1^ ^2^ This pattern of spending is especially pronounced for people with cancer. Despite the cancer trajectory being highly predictable, costs escalate at an exponential rate up to the time of death,^3^ with the additional costs almost entirely attributable to an increased use of acute hospital services, in particular emergency department (ED) visits and unplanned hospital admissions.^1^ ^4^

The increased use of acute hospital services towards the end-of-life would not be such a concern if it improved outcomes for patients with cancer and their families. However, evidence suggests this is not the case. Instead, prolonged hospital admissions and/or multiple ED visits in the last month of life are associated with greater physical distress, overall dissatisfaction with care and more than a threefold increase in the likelihood of psychiatric illness among bereaved relatives.^5–7^ Furthermore, for the majority of people with cancer, acute hospital care is diametrically opposed to their stated preferences for end-of-life care.^8^ Most (64–84%) people with cancer prefer to be cared for and die at home,^9^ surrounded by their loved ones and free from the stressful environment of an acute hospital.^9–12^

Reducing patients with cancer use of acute hospital services towards the end-of-life therefore provides an opportunity to improve overall care quality and reduce healthcare costs. These clear individual and societal benefits have motivated policymakers to introduce measures to minimise acute hospitalisations. To date, however, the impact of such initiatives has been limited; instead, the number of people with cancer experiencing multiple ED visits and/ or with prolonged hospital admissions towards the end-of-life has risen.^13^ ^14^ In England, most ED visits represent self-presentations.^15^ Hence, if future initiatives are to be successful, a more comprehensive understanding of *why* people with cancer choose ED care is required. Only then will it be possible to devise a system of end-of-life care services that can effectively serve the needs and preferences of people with cancer and their families.

Most of the existing research on end-of-life ED use by people with cancer has focused on quantifying attendance and/or identifying factors associated with an increased risk of multiple visits in the last month of life.^16–18^ While these studies have identified a number of sociodemographic, environmental and clinical risk factors (eg, sex, age, ethnicity, socioeconomic status and type of cancer), evidence for *why* people with cancer decide to attend the ED is limited.^19^ ^20^ In order to address this issue and help guide development of future healthcare services, we conducted the following qualitative study. The aim of our study was to explore the decisions of people with advanced cancer and their caregivers to seek ED care, and understand the issues that influence the decision-making process.

## Methods

This study is reported following the consolidated criteria for reporting qualitative studies (COREQ).^21^

### Theoretical framework

Many previous studies have explored patients’ use of healthcare services^22–25^ and extant models of health-seeking behaviour can be useful to guide future research and investigation. The most widely acknowledged theory of healthcare usage is the ‘Behavioral Model of Health Services Use’ developed by Andersen in 1968 and subsequently published with Newman in 1973.^22^ Although initially developed to explain non-discriminative healthcare use among the general population, the model has since been applied to a variety of services and populations.^26^ It has not, however, been used to examine the uptake of healthcare services by people with advanced cancer or at the end-of-life. In 1992, Padgett and Brodsky^27^ modified the model, specifically to explain non-urgent ED use. In this adapted version, three stages of decision-making are identified: (1) problem recognition; (2) decision to seek medical care; and, (3) decision to use the ED. Predisposing, enabling and need-based factors—as per Andersen and Newman's original model—are proposed to influence each of these three stages.^27^ This modified version of the model was used as a framework for our study. The model's utility when applied to a different population group—people with advanced cancer—was also tested.

### Setting

A large university teaching hospital in south-east London, serving an ethnically, socially and economically diverse urban population of approximately two million. The hospital's ED sees over 120 000 patients each year—about 350 patients a day.^28^

### Participants

Participants were adults (≥18 years) with advanced cancer who had recently attended, from their private residence, the hospital's ED; and where applicable their main caregiver (see box 1).

Box 1 **Study eligibility criteria**

Inclusion criteria: patients

- Adults (≥18 years).
- Diagnosed with advanced cancer by a qualified healthcare professional involved in the patient's care. Advanced cancer defined as cancer that has invaded surrounding body tissues and/or metastasised, and is not curable and is life-threatening.
- Assessed as having a prognosis of weeks to short months by a qualified healthcare professional involved in the patient's care.
- Attended, from their private residence, the emergency department (ED), within 2 weeks of screening for the study.

Inclusion criteria: caregivers

- Adults (≥18 years).
- Identified as their caregiver by an eligible patient recruited to the study. Caregiver defined as an unpaid family member/close friend involved in caring for the patient's physical, emotional and/or practical needs.

Exclusion criteria: patients and caregivers

- Participants incapable of providing informed consent.
- Patients attending the ED from nursing homes, care homes or other institutionalised care settings.
- Patients brought to the ED by representatives of Her Majesty's Prison Service and under their supervision.
- Participants whose clinical team considers them to be too unwell and/or distressed to participate in the study.

Recruitment of patients was through the hospital's palliative care and acute oncology teams. Between 16 December 2014 and 31 July 2015 both teams screened all new referrals against the study's eligibility criteria. The acute oncology team also screened all ED discharges for people with advanced cancer who attended the ED but were not admitted. Any eligible patients identified were first approached by a clinical member of the team who provided them with a leaflet about the study and assessed their interest in participating (patients already discharged were phoned at home). Those who expressed interest in the study were then followed-up by a member of the research team, either face-to-face or via the telephone.

Recruitment to research studies can be especially challenging in vulnerable population groups such as those with advanced diseases. Issues such as gate keeping, high symptom burden and a rapidly changing clinical picture often result in poor recruitment and/or high attrition rates.^29^ To help overcome some of these challenges the research and clinical teams collaborated closely during the study period, with face-to-face meetings at least twice weekly. This enabled prompt follow-up of potential participants, most of whom were contacted by the research team within 24 hours of them expressing interest in the study. Additional strategies to reduce attrition included flexibility around the interview setting and timing, as well as the option to conduct joint patient and caregiver interviews if preferred.

We adopted a maximum variation (heterogeneity) sampling strategy to identify people with a range of characteristics and capture potentially richer and more diverse data relevant to the research question.^30^ Sampling criteria were based on the findings of a recently conducted systematic review exploring factors associated with ED attendance by patients with cancer in the last month of life, and were: sex; age; ethnicity; socioeconomic status; type of cancer; and use of palliative care services.^18^

Caregivers were identified through patients enrolled to the study, all of whom were asked if they had a family member/close friend that helped care for any of their physical, emotional and/or practical needs. For patients who identified a caregiver, permission was sought for a member of the research team to approach the caregiver regarding study participation.

Recruitment of both patients and caregivers continued until data saturation was achieved. Specifically, this was the point when we were confident that the emerging themes and constructs appeared to be fully represented by the data collected. Additional interviews did not result in a greater depth of understanding or the generation of new themes and/or constructs.^31^

### Interviews

Each participant consented to a one off semistructured interview with researcher LH (palliative care physician (MBBS, MRCP) and PhD clinical training fellow; female). All interviews were audio recorded and field notes were made during or immediately after each interview. At the request of participants, caregiver interviews were conducted jointly with patients apart from in one case where the caregiver interview occurred immediately following the patient interview. During the consenting process LH explained that she was working with the palliative care or acute oncology team to conduct a study about people's decisions to seek ED care. No further information about the research team was offered.

During interviews participants were asked to describe the most recent time that they, or their family member/close friend, attended the ED and the issues that influenced their decision-making process. In order to enhance the consistency and completeness of data collected across cases, topic guides were developed (based on the study's theoretical framework^27^), piloted and used during interviews (see online supplementary file 1—patient interview topic guide; online supplementary file 2—caregiver interview topic guide). Participants were encouraged to talk in-depth about their thought processes with prompts used to elicit further information when required.

10.1136/bmjopen-2016-012134.supp1 supplementary file

10.1136/bmjopen-2016-012134.supp2 supplementary file

### Analysis

Interviews were anonymised, transcribed verbatim and analysed using a constant comparative approach.^32^ Analysis began with open coding of the transcript where meaningful words, phrases and statements were identified, followed by more detailed axial coding as items emerged. These items were then grouped into themes which became further refined as the analysis continued. Differences and similarities were explored within and across interviews. Where new themes emerged, earlier interviews were reanalysed to consider further and/or alternative meaning, with particular attention paid to non-confirmatory/divergent cases.

All interview transcripts were analysed by researcher LH. To address issues of analytical rigour, credibility and trustworthiness, a selection of interviews were also analysed by researchers CE-S and JK, and then reviewed with LH. Where coding differed, areas were reconsidered and discussed until consensus was reached regarding interpretation and overall meaning.

## Results

### Characteristics of participants

**Figure 1:**
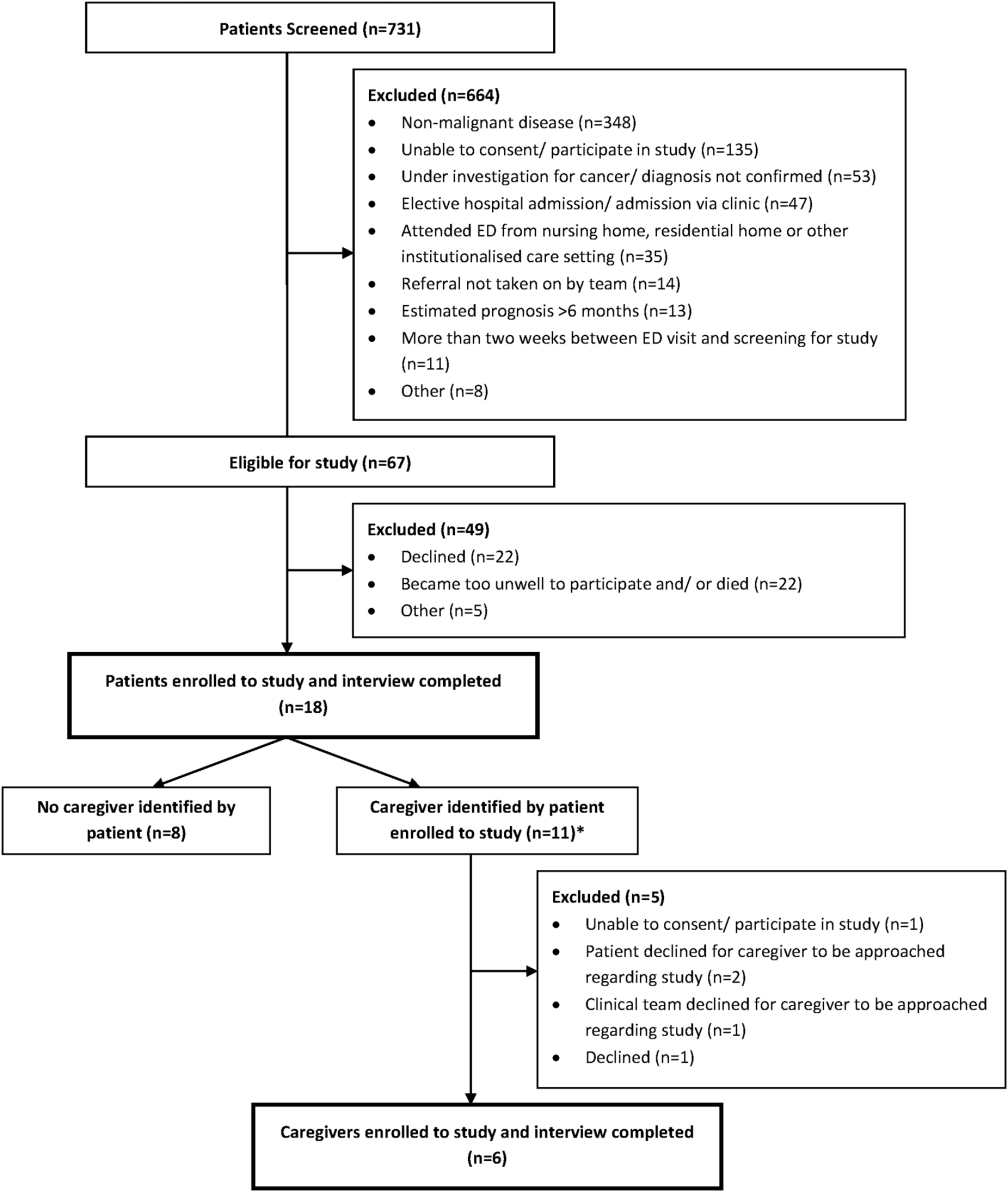
Flow diagram of patient and caregiver recruitment. *One patient identified two family members. ED, emergency department.

Seven hundred and thirty-one patients were screened for the study, of whom 67 met the eligibility criteria and were approached regarding participation. Twenty-two patients declined to participate citing reasons that included ‘not interested’ and ‘didn't feel up to it’. A further 22 patients became too unwell or died in the short time period between being approached about the study and an interview being arranged. The final five patients were excluded for other reasons, including closure of a hospital ward because of a Norovirus outbreak. A total of 18 patients were recruited to the study (figure 1).

Among the 18 patients recruited, 10 identified a family member/close friend as their main caregiver, with one patient identifying two family members. Permission was given for eight of these individuals to be approached about the study. One caregiver declined to participate stating ‘they didn't have enough time’ and one was unable to consent; the remaining six were enrolled to the study (figure 1).

No participants withdrew from the study, however two interviews ended early. In one case (ED03), after 27 min, the patient felt unwell and unable to continue with the interview. In the second case (ED16), the patient found the study questions frustrating, in particular the level of detail being asked, and requested to stop after 13 min. Eighteen of the 24 interviews were conducted in hospital, 5 in participants’ homes and 1 in the hospital's Macmillan Information and Support Centre. No other persons apart from those involved in the study were present during the interview process. Interviews lasted an average of 31 min (range 13–57). The mean number of days between the patient's interview and death was 90 (range 7–252) (Five patients remained alive as of the 16 February 2016).

Characteristics of the 18 patient participants are presented in table 1.

**Table 1:** Characteristics of patient participants

Type of cancer	N	Sex (male/female)	Age in years (mean (range))	Ethnicity (white British/other)	Socioeconomic status* (1–2/3–5)	Under community palliative care prior to ED visit (yes/no)	Reasons for ED attendance
Lung	4	3/1	70 (45–86)	3/1	3/1	1/3	Focal seizures; malaise; pain; breathlessness and malaise.
Haematological malignancies	4	2/2	75 (59–90)	4/0	4/0	1/3	Pain; rectal bleeding; fever; fever.
Prostate, gynaecological and urinary tract	4	2/2	72 (55–88)	3/1	4/0	2/2	Fall; pain; facial weakness and malaise; haematuria.
Gastrointestinal and hepatocellular	3	1/2	59 (42–68)	1/2	2/1	1/2	Pain; pain fever and cough; fever.
Other	3	1/2	37 (19–47)	0/3	1/2	0/3	Pain; facial numbness and headache; fever and pain.

*Socioeconomic status derived from index of multiple deprivation (IMD) quintiles (1st—most deprived; 5th—least deprived). The IMD is an area based measure of deprivation that uses Lower Super Output Area geography to compare deprivation between neighbourhoods in England.

ED, emergency department.

### Accounts of the decision-making process

During each interview participants narrated their own unique account of the events leading up to their ED visit. In keeping with the study's theoretical framework,^27^ participants’ overall decision-making was composed of three key stages: (1) problem recognition; (2) decision to seek medical care; and, (3) decision to use the ED. For some participants these decision-making stages occurred quickly (within minutes), while for others one was deliberated to a greater extent than the other. For a few participants the initial ‘problem recognition’ and ‘decision to seek medical care’ stages were so intuitive that they struggled to recognise any decision-making at this time. For example, patient ED16 began his interview by describing back pain he had experienced in the days leading up to his ED visit. When asked why he decided to seek medical care, he struggled to describe his decision-making further, instead repeating that pain was the reason he sought help.

ED16 [patient]: Well the pain. Researcher: Okay, but you'd had it [the pain] for a few days? ED16: Yeah. Researcher: So what changed? ED16: Well the pain.

Later during the interview ED16 was able to elaborate further. He explained that over a period of days his pain got progressively worse to the extent that on the morning of his ED visit he had struggled to get out of bed. It was this feature—the pain limiting his mobility—that triggered his decision to seek medical care.

Each of the decision-making stages, and the issues that influenced them, are presented below.

### Stage 1: problem recognition

All participants described physical problems during their interviews with a wide range of symptoms reported, including pain, fevers, breathlessness and seizures (table 1). While most reported these physical symptoms as central to their decision to seek medical care, it became apparent that most experienced symptoms at many other points in time for which they did not decide to seek help. Instead it was participants’ perception, or interpretation, of their symptom(s)—rather than the symptom per se—which appeared to influence their decision-making.

Symptom interpretation varied considerably between participants. Some interpreted their symptom(s) as severe and felt compelled to seek medical care as soon as possible. Others perceived their symptoms as mild, or to be expected, and consequently did not decide to seek medical care until another event triggered them to seek help. Three concepts emerged as influencing participants’ symptom perception/interpretation: (1) anxiety relating to their underlying cancer diagnosis; (2) prior symptom experiences; and (3) education and knowledge.

#### Anxiety relating to underlying cancer diagnosis

A number of participants conveyed narratives explaining how their diagnosis of cancer felt like a ‘death sentence’ and was ‘always on their mind’. Any new symptom experienced would be interpreted within this context. For example, patient ED02, a woman with colorectal cancer, described how she immediately thought her cancer was progressing when she developed pain.

ED02 [patient]: it's always going to trigger (Researcher: Okay) is this thing growing? Is this get…is it getting out of hand you know? (Researcher: Okay) You know, what is, what is going to happen?

Two patient characteristics appeared to influence participants’ anxiety of their cancer: age; and religious or spiritual beliefs. Compared to younger patients, older patients tended to describe less anxiety related to their cancer diagnosis and subsequently were less likely to perceive a new symptom as always being cancer related.

Researcher: So when you fell onto the floor, that [cancer] wasn't something going through your mind?

ED06 [patient]: When, when?

Researcher: When you slipped from the chair?

ED06: Oh! Good God no! I was looking at the bloody football score [laughs].

Participants’ with religious or spiritual beliefs described how their faith helped them cope with their cancer diagnosis and any symptoms they experienced.

ED18 [patient]: …my faith is very strong in in what I believe, and, that really takes care of a lot of the…the burden if I should say, (Researcher: Okay) you know. When…when I get to a state where I get like a bit of a wimp, I pray.

#### Prior symptom experience

Participants’ recollections of previously experienced symptoms also influenced how they interpreted their situation. Many considered a new symptom as ‘severe’ and/or ‘urgent’ if it was similar or related to symptoms they had experienced around the time of their cancer diagnosis. This was illustrated by patient ED10 who explained how he had been diagnosed with metastatic lung cancer after having a grand-mal seizure. Despite experiencing many other symptoms since then, ED10 had not had another seizure until the week of his interview when he developed a partial seizure of his arm and decided to immediately seek help. ED10 described his thoughts at this time:

ED10 [patient]: That decision came because of the past experience. So we know its brain. (Researcher: Okay) So our fear was it's—it may have grown bigger and the pressure could be you know imminent danger.

#### Education and knowledge

Several patients had received advice from healthcare professionals regarding specific symptoms. This education and knowledge influenced their interpretation of how important certain symptoms were and whether or not they decided to seek help.

ED12 [patient]: …the reason I came in is because erm, I've got cancer, and erm, I was erm being looked after at XX Hospital team and they told me, erm if I've got a temperature above erm I think it's 37 point something then I should go to my nearest A&E. I did have a temperature of 38 point. I called them and the nurse said to me I should make my way here just in-case I had an infection.

The levels of anxiety relating to having a diagnosis of cancer was variable across individuals, as above, and did not appear to differ between those who were and were not receiving community palliative care. Participants who received symptom advice/information from their palliative care team did describe less anxiety regarding new symptoms and several also reported seeking alternative sources of help before deciding to attend the ED.

### Stage 2: decision to seek medical care

For those who interpreted their symptom(s) as severe, their decision to seek medical care followed rapidly and was often hard to separate from the initial problem recognition stage. However, for participants who did not seek help immediately and instead accommodated or managed their symptom(s), this second decision to seek medical care appeared to occur later when for some reason they were no longer able to tolerate, or accommodate, their symptom(s). Reasons for why this accommodation broke down included situations where the symptom changed in character, started to interfere with activities of daily living and/or persisted beyond an arbitrary time threshold. Patient ED25, a woman with bladder cancer, explained how despite having experienced multiple previous episodes of cancer-related haematuria, she decided to seek help for the most recent occurrence because the bleeding became increasingly severe and persisted beyond 3 days.

ED25 [patient]: the bleeding just got heavier and heavier and heavier and went on for about four days and didn't abate at all—it just got worse.

In another example, patient ED18, a woman with metastatic breast cancer, described how she had been managing with back pain for several days. However, it was when the pain became so severe that is started to restrict her movements that she decided she needed help.

ED18 [patient]: So it's like certain movements I couldn't even do. (Researcher: Okay) Yeah, it like just trapped me there.

Sanctioning by family and/or healthcare professionals was also described. During one interview, patient ED12 explained how her family would encourage her to seek medical care for symptoms that she felt she was coping with. At times their insistence was so great it would lead to her seeking medical care.

ED12 [patient]: Well they put pressure on me and sometimes to shut them up [laughs], just to shut them up, I would call the nurse, yes they [family]…they…they influenced me to call the nurse then yeah. To keep them [family] happy you know and to stop nagging me.

### Stage 3: decision to use the ED

Once participants had decided to seek medical care, their decision to use the ED was explored. Four concepts emerged as key to this stage of decision-making: (1) availability and ease of access; (2) hospital facilities and environment; (3) trust and healthcare provider continuity; and, (4) ability to abdicate responsibility.

#### Availability and ease of access

Both the availability of healthcare services and their ease of access were important to participants when deciding where to seek help. Participants preferred services where they could receive care quickly and with little stress or inconvenience. Overly complicated systems were bypassed for more straightforward options, for example, patient ED01 described how he chose to attend the ED over an alternative healthcare service because access to the latter often involved multiple steps and time. By comparison, once he arrived at hospital, healthcare professionals would come to him and the responsibility for identifying and accessing the ‘right’ care was organised for him.

ED01 [patient]: …they would have to go through someone else to go through someone else (Researcher: Mmm) do you know what…I wouldn't want anything like that. Erm or I might as well just come to hospital in that case (Researcher: Okay) because eventually I'll be in a safe place and they'll come to me.

Trouble accessing appointments, especially those which were urgent and/or out-of-hours, was an important barrier to participants’ seeking help from their general practitioner (GP) and often facilitated their decision to instead attend the ED.

ED08 [daughter]: Can't get hold of dad's GP of a weekend. (Researcher: Okay) (ED07 [patient]: No) I can't get hold of my GP in XX [local area of patient] of a weekend so erm (Researcher: Okay) it goes through to XX [out of hours service].

A few participants described contacting other healthcare services prior to attending the ED, in most cases telephoning their oncology nurse specialist or community palliative care team. When probed further about these decisions only one participant described calling because she hoped this would result in some action that could help her avoid attending the ED. The remaining participants reported other reasons for calling their oncology team. One patient explained that he called his oncology team as a courtesy; he had been advised to call them with any new symptoms. Another patient explained how calling the oncology team sometimes expedited the hospital's triage process, stating:

ED22 [patient]: Yes and the advantage of phoning ahead is they sort of expect you, and therefore you might get through a stage quicker.

Community palliative care services were often not called as participants felt they would be unable to help in an emergency situation. Instead community palliative care services were described as being able to help with non-urgent issues and help facilitate communication between services such as their GP and oncology.

In only one case was advice from the patient's GP sought prior to them attending the ED. During this interview, ED17, son of ED16, explained that since his father's cancer diagnosis his GP would always make himself available, even if his schedule was full.

ED17 [son]: He's even gave him an open appointment that if we need to see him we will see him. If the…if the reception says there's not a…a…a space in the normal appointment times, he makes time at the end of the surgery.

This level of GP support meant that both ED16 and ED17 felt they would always seek advice from their GP prior to seeking help elsewhere; ED16 explained that although he thought his father needed to be in hospital he still decided to contact his GP first.

ED17 [son]: He [GP] would come at the end of the surgery……Although to us at the time he needed to be in there [hospital]. (Researcher: Okay) But as I…as I say it's…it's easier going through the GP.

#### Hospital facilities and environment

When deciding where to seek help from, participants tended to favour care delivered in a hospital over other less acute or community settings. They described feeling comforted by the frequent monitoring of their condition and the presence of healthcare professionals.

ED19: …Like in the hospitals when you go they give you lots of attention, lots of treat—you are under their eyes, they come and check you, monitoring you.

Several participants also described how the hospital provided facilities and equipment that they considered essential for the management of their symptoms. Many held strong beliefs about the type and level of care their condition required, for example, intravenous antibiotics, which appeared to originate from a combination of their clinical knowledge, previous healthcare experiences and/or an instinctive feeling regarding the treatment they required. None of the participants interviewed identified alternative settings where inpatient care could have been accessed. Patient ED10 explained how his decision to call 999 was based on both his previous symptom experiences as well as his knowledge of his cancer.

ED10 [patient]: …Because I know what's going on. I have a slight idea, I have the fear that this could be this, because we've done a lot of research, (Researcher: Okay) on how things work. And now I'm on a few forums as well and so I know cases where—what other people have experienced.

In comparison, patient ED04 reported instinctively ‘knowing’ that her symptoms required hospital care despite not having any specific treatments or tests in mind. She said:

ED04 [patient]: Well because I knew he [GP] couldn't do anything about it but get me to…to the hospital because of all that, and I knew. He wouldn't—they couldn't have done anything, only getting the ambulance and coming here.

#### Trust and healthcare provider continuity

During interviews several participants spent time talking about their relationship with the hospital, which often began around the time of their cancer diagnosis. Participants described how over months, or even years, of investigations and treatment, relationships with hospital professionals had developed. For many this process had led to them becoming familiar with the hospital and their clinical team. Many held feelings of trust or ‘belief’ in the care being provided by the hospital.

ED05 [daughter]: It's…it's nice to come to a place that you're not frightened of. (Researcher: Yeah) You know, that makes you feel good.

ED04 [patient]: That's right you believe in you know, you've got to believe in it.

ED05: Yeah we believe—that's the word mum we believe in XX [hospital] don't we?

ED04: We believe in it—first words are don't take me anywhere (ED05: Yes) but XX [hospital]. (Researcher: Okay) That is true.

In contrast to the hospital relationships that had developed, most participants described rarely seeing their GP during this time. Some stated explicitly that their GP had little or no role in the management of their cancer.

Researcher: …do you tend to see your GP more now or less now? How do you feel?

ED13 [patient]: Never see him.

ED14 [wife]: Yeah, very rarely.

ED13: Never see GP. Don't see the point.

When it came to seeking help, these relationships influenced participants’ decision-making, especially at times of crisis when participants defaulted to services they had previously used and felt safe with. In the majority of cases this ended up being the hospital—only one patient described having a more trusting relationship with their GP than with the hospital.

#### Abdicate responsibility

When participants decided to attend the ED many also described acute feelings of being unable to cope or manage at home.

ED25 [patient]: Because my big fear was—would be that, you know…a giant clot is gonna form and I'm gonna be yelling and screaming in somebody's house or restaurant get me to hospital you know.

By seeking help from the hospital participants were enabled—and in many cases expected—to abdicate responsibility for their care to healthcare professionals. The carer of one patient explained how she brought her father to the ED because she had run out of options to care for him at home.

ED08 [daughter]: Erm, and what am I supposed to do if that happens? I'm not qualified in anything other than just sort of being able to hold him tight (Researcher: Mmm) and cuddle him.

## Discussion

This qualitative study provides new findings that help explain *why* and *how* people with advanced cancer decide to seek ED care. We have identified individual's symptom interpretation, their prior patterns of health-seeking behaviour, feelings of safety and familiarity with the hospital setting and difficulties accessing community healthcare services as important issues influencing the decision-making process.

**Figure 2:**
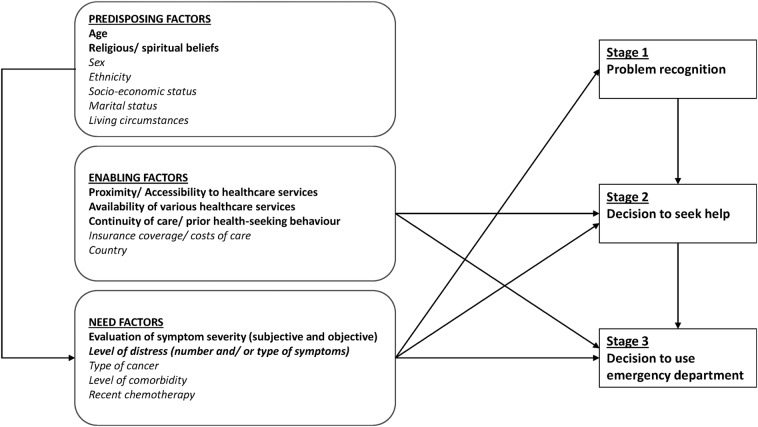
Model of factors influencing advanced cancer patients’ emergency department use. Factors in bold indicate those with evidence from current study, factors in italic indicate those identified from previous studies.

Consistent with our study's theoretical framework, participants described a three-stage process of decision-making. The influence from predisposing, enabling and need-based factors, did however vary from the original framework, as illustrated in figure 2.

Need-based factors were identified as the most important influence on patients’ problem recognition (stage 1). In particular, patients’ anxiety relating to their underlying cancer diagnosis significantly influenced their symptom perception in terms of meaning and severity. While predisposing factors, such as age, also influenced problem recognition, this effect appeared to act through patients’ symptom perception/interpretation. For example, we found older patients were less likely to interpret symptom(s) as a sign of illness and therefore less likely to recognise them as a problem. A number of previous studies have identified variation in patients with cancers’ ED attendance based on differing sociodemographic (predisposing) factors.^16–18^ Our findings, however, suggest that rather than these factors per se influencing patients’ ED use, it is the variation in symptom perception among these groups that ultimately determines the overall differences seen. This mechanism of action is further supported by previous studies that have identified variation in symptom perception by patient sociodemographics, including differences found across social class^33^ and ethnicity.^34^ Addressing the anxiety and other psychological sequelae commonly experienced by patients with cancer is an important component of high-quality holistic care. Evidence that people experience an increasing sense of vulnerability and/or lack of control prior to seeking emergency hospital care has been reported in similar studies.^19^ Integrating interventions to reduce anxiety and/or enhance coping to current end-of-life support services may be one approach towards modifying patient's symptom perception/interpretation. Understanding these decision-making mechanisms is important for clinical practice, especially at a policy level where the findings may be used to inform services delivery and/or intervention development. We suggest that rather than developing policies/interventions that target a particular ‘high-risk’ patient group, for example, ethnic minority patients or those of lower socioeconomic status, educating patients regarding end-of-life symptoms is likely to be more effective through addressing the issues of symptom interpretation and/or levels of distress. Indeed targeting patients identified as having greater levels of anxiety regarding their symptoms may be more effective and not exclusive to those with specific predisposing factors.

While there was strong evidence for the influence of need-based factors on patients’ problem recognition (stage 1), our study did not support enabling factors as also being influential. These were however, important to both subsequent stages of decision-making: decision to seek help (stage 2); and, decision to use the ED (stage 3) (figure 2). In healthcare research enabling factors are arguably the most important to consider since they represent the group of variables most amenable to change. Understanding how this group influences patients’ health-seeking behaviour can therefore provide policymakers with better evidence to develop and/or modify existing healthcare structures to improve patient outcomes. Presently, in the UK, one in every two people will be diagnosed with some form of cancer during their lifetime. Despite advances in oncology care and treatment, 50% of those diagnosed will ultimately die from their disease.^35^ For these patients, the current model of healthcare delivery is one where as their disease progresses they transition from receiving exclusively oncological care—a predominantly hospital-based specialty, to mostly palliative care—more community-based.^36^ ^37^ Implementing this model of transition is, however, challenging. Studies have found that many oncologists are reluctant to refer their patients to palliative care which some perceive as ‘an alternative philosophy of care incompatible with cancer therapy’ (Schenker *et al*, 2014, pp. e41).^38^ Furthermore, inaccurate prognostication often leads to an overestimate of survival,^39^ meaning that many transitions to palliative care are often initiated too late in a patients’ illness or do not happen at all.^40^ During interviews we observed that patients’ health-seeking behaviour tended to favour hospital-based care. This preference occurred in part as a result of the extensive hospital contact patients had experienced earlier in their illness, along with very limited GP and community service engagement during this time. Patients require time to become familiar with new services and for their patterns of health-seeking behaviour to change. Studies showing an association between earlier palliative care referral and fewer ED visits at the end-of-life,^41^ ^42^ as well as those that show less aggressive end-of-life care with greater community healthcare contact^43^ further support these findings. If the time between palliative care referral and patient death is insufficient, patients are likely to continue to use services they are familiar with, especially at times of crisis. New models of healthcare delivery that encourage earlier integration between oncology and palliative care are required to address this issue.

The availability of community healthcare services was also important in patients’ decision-making, with several participants describing having ‘no alternative’ to attending the ED. In a recently published qualitative critical incident study of people with advanced respiratory disease, Karasouli *et al*^19^ found that the decisions of participants to seek emergency hospital care were reinforced in those who had experienced difficulty accessing support from community services. While access remains critical, we found that the structure of community services also needs consideration. Our study highlighted key features of the hospital environment described as important to participants, for example, many felt reassured by the presence of healthcare staff to whom they were also able to abdicate responsibility. Community services need to develop in a way that allows them to meet such preferences as expansion of existing services alone may not necessarily translate into reduced acute hospital service use. Increasing the number of inpatient hospice beds may be one possible solution.

### Limitations

There are limitations to this study. As with all qualitative research it is possible that our findings were influenced by the researcher's personal biases and/or experience. We attempted to address this by using a maximum variation sampling strategy and performing dual coding for a selection of interviews. Although member checking of the interview transcripts and/or study findings could have further enhanced the rigour of our results, this was unable to be performed due to the rapid deterioration of many of the participants.

The setting (London) of our study is likely to have influenced some of our findings. Compared to other more rural settings patients in London have greater access to acute hospital care. Community healthcare services are also known to vary by region. Some of our study findings may therefore not be applicable to people living in different environments, especially those in more rural settings.

We also only interviewed patients who had decided to seek ED care meaning that the decision-making process of those who used alternative services was not explored. Future research exploring whether the issues identified remain relevant to patients who choose community services would provide further insight and understanding of this topic.

Finally, it is important to acknowledge that for many people acute hospital care does not represent an adverse event. In many situations the ED is the most appropriate setting for urgent care needs to be investigated and managed, and the importance of providing individualised patient-centered care, including ED care if needed, should not be overlooked.

## Conclusions

Drawing on Padgett and Brodsky's modified version of the ‘Behavioral Model of Health Services Use’, this study provides new evidence for *why* and *how* patients with advanced cancer decide to seek ED care. Issues influencing the decision-making process included: (1) disease-related anxiety; (2) prior patterns of health-seeking behaviour; (3) feelings of safety and familiarity with the hospital setting; and, (4) difficulties accessing community healthcare services—especially urgently and/or out-of-hours. These insights provide healthcare professionals and policymakers with a greater understanding of how systems of care may be developed to help reduce ED visits made by people with advanced cancer. In particular, our findings suggest that the number of ED visits could be reduced with greater end-of-life symptom support and education, earlier collaboration between oncology and palliative care and with increased access to community healthcare services.
