# Supplementary material for: ‘I'll be in a safe place’: a qualitative study of the decisions taken by people with advanced cancer to seek emergency department care
Source: BMJ Open. 2016 Nov 2;6(11):e012134. doi: 10.1136/bmjopen-2016-012134 (PMC5129048; doi:10.1136/bmjopen-2016-012134)
Supplement: supplementary file [file bmjopen-2016-012134supp1.pdf]

Topic Guide: Patient

How do patients with advanced cancer decide to attend the emergency department, and what influences their decision-making at this time? A qualitative case study

Aim: To investigate the processes by which advanced cancer patients, and their caregivers, decide to attend the emergency department, and; to explore advanced cancer patients', and their caregivers', preferences for an urgent care service.

Introduction:

- Introduce researcher and study purpose.
- Thank participant for taking part.
- Explain that this is an opportunity for the participant to tell the researcher, in their own words, why they decided to attend the ED and how the decision to go to the ED came about.

Administrative Tasks:

- Describe the interview process, including the following:
    - The interview will be audio recorded.
    - All information from the interview will remain confidential unless there are concerns regarding the safety of the participant, others, and/ or information is disclosed that is required to be reported by law.
    - Expected interview duration is approximately 60 minutes.
    - The participant can stop the interview at any time and/ or decline to answer any questions asked.
    - There are no right or wrong answers.

- Encourage the participant to speak freely throughout the interview. Reassure participant that any names or identifying information disclosed will be removed when the interview is transcribed.

- Make sure the participant understands that the interview is regarding their most recent ED visit: probe participant for the date/ time of patient's most recent ED visit. Check that this is consistent with study records.

Check Written Consent Form

-- START RECORDING --

Icebreaker:

	Examples
1. General question aiming to put participant at ease	**"Tell me a little bit about yourself, and who the key people are in your life?"** ***OR*** **"How did you come to be in your current situation?"**

Cancer Background

Topic Areas to be Covered	Examples
1. Explore background to cancer diagnosis • What type of cancer? • When were they diagnosed? • What is the current situation regarding their disease status?	**"Can you tell me about your diagnosis of cancer?"** **OR** **"Can you tell me about when you were first diagnosed with cancer and what has happened since then"** <br>**"How is your health at the moment?"**

ED Attendance:

Topic Areas to be Covered

1. Explore the events surrounding the patient's ED attendance
   - Where was the patient?
   - Were they alone?
   - How did they get to the ED?
   - Did they go to the ED alone or with someone else?

Examples

"I'm interested in knowing more about the time that you came to the ED. What can you tell me about that day?"

OR

"Talk me through what happened that day."

Explore Decision Making:

Topic Areas to be Covered

1. Explore the decision to go to the ED
   - Who made the decision to go to the ED?
   - Who else was involved in making the decision?
   - Were there differences in opinions?
   - How long did it take to make the decision?

Example Questions

"Explain to me how the decision to attend the ED came about?"

"Talk me through your thoughts during this time."

2. Explore influencing factors in decision making
   - What were the factors?
   - What made them important?
   - If not mentioned by participant, ask specifically whether any of the following factors influenced decision making:
     a. **Symptoms** experienced by the patient;
     b. **Access** to healthcare services (both physical in terms of location of patient to the service and non-physical in

        terms of the availability of such

"What things did you weigh-up when deciding whether or not to go to the ED?"

"You've told me about these important factors that influenced your decision-making [list factors mentioned]. Explain to me why they were important and how they influenced your decision?"

service);

c. **Religion**;

d. Previous **end of life discussions or advance care planning**;

e. **Previous advice** or guidance provided about healthcare services and utilisation;

f. **The set-up at home** (e.g. living alone? Married? Children in the house); and

g. **Previous patterns of consulting behaviour** and **experiences of healthcare services** (including both the ED and elsewhere)

3. Explore hierarchy and relationship between factors mentioned<br>• Was there one factor that was the most important?<br>• Were there factors that were not so important?<br>• Were they related?	**"Were any of these factors you've mentioned more important than others? In what way?"**<br><br>**"Do you think any of these factors were related to each other? In what way are they related? Or are they separate things that you considered individually?"**

Experience at ED and reflecting on decision:

**Topic Areas to be Covered**	**Examples**
1. Explore ED experience further	**"Tell me a bit about what happened when you arrived at the ED?"**

2. Explore previous healthcare utilisation behaviour — **"Have you been that unwell before?"**
   - Have there been similar situations in the past?
   - Did they act the same or differently?

3. Ask participant to reflect on decision — **"Looking back now, what are your reflections on the experience and decision that was made?"**
   OR
   "If the same situation arose again, would you do anything differently? What would you do differently?"

4. Explore preferences for acute care services — **"If you needed urgent care again, can you describe to me what you would consider an ideal service?"**
   - Would they prefer care at home or in hospital?
   - Why?

Concluding Interview

1. Remind participant about the study purpose — **"The purpose of this study is to try and understand how people with cancer, and their family or friends decide to go to the ED. Are there any areas that we haven't talked about that you feel would be important to discuss?"**

2. Ask for any final thoughts or comments — **"Do you have any questions, final thoughts or comments?"**

-- STOP RECORDING --

--------------------------------------- COMPLETE PARTICIPANT DEMOGRAPHICS FORM -------------------------

Final Tasks:

- Check participant is okay with the interview process and enquire if they would like any additional support at this time. If participant shows any signs of distress refer to Distress Protocol for further action.
- Leave details of the Macmillan Information & Support Centre, based at the Cicely Saunders Institute, King's College Hospital.
- Ask patient if they would like a copy of the study findings sent to them.
- Thank participant.
